# Determinants of Weight Gain Among Adult Tuberculosis Patients During Intensive Phase in East Gojjam Zone Public Health Institutions, Amhara Region, Ethiopia. A Cross‐Sectional Study

**DOI:** 10.1155/jnme/6654681

**Published:** 2026-06-26

**Authors:** Dires Delessa Alemu, Getnet Gedif, Habitamu Mekonen, Mequanente Dagnaw, Helen Asmamaw Asres, Nakachew Mekonnen Alamirew, Yilkal Tafere

**Affiliations:** ^1^ Department of Public Health, College of Medicine and Health Sciences, Debre Markos University, Debre Markos, Ethiopia, dmu.edu.et; ^2^ Department of Public Health Emergency Management, Amhara Public Health Institute (Debre Markos Branch), East Gojjam Zone, Debre Markos, Amhara Region, Ethiopia; ^3^ Department of Human Nutrition, College of Health Science, Debre Markos University, Debre Markos, Ethiopia, dmu.edu.et; ^4^ Department of Epidemiology and Biostatistics, Institute of Public Health, University of Gondar, Gondar, Ethiopia, uog.edu.et; ^5^ Department of Medical Biotechnology, Institute of Biotechnology, University of Gondar, Gondar, Ethiopia, uog.edu.et

**Keywords:** Amhara region, East Gojjam, Ethiopia, intensive phase, TB patients, weight gain

## Abstract

**Background:**

Ethiopia ranks among the countries with the highest tuberculosis (TB) burden globally. Weight gain during the intensive treatment phase is a reliable indicator of successful TB treatment outcomes; however, evidence on its magnitude and its determinants in Northwest Ethiopia, particularly, in East Gojjam Zone, remains limited.

**Objective:**

This study aimed to determine the magnitude of weight gain and its determinants among adult TB patients during the first two months of the intensive treatment phase in East Gojjam Zone public health institutions, Northwest Ethiopia, 2025.

**Methods:**

An institution‐based cross‐sectional study design was conducted from June 4 to August 4, 2025. Using proportional simple random sampling, 619 adult TB patients aged 18+ years under follow‐up at public at 31 health institutions were enrolled. Data were collected via interviewer‐administered questionnaires and medical record reviews. Binary logistic regression identified determinants factors; variables with *p* < 0.25 in bivariable analysis were entered into multivariable logistic regression analysis.

**Results:**

Weight gain was achieved by 428 (69.1%) of TB patients (95% CI: 65.4, 72.7) during the intensive phase treatment. Six factors were independently associated with weight gain: completed drug adherence (AOR: 4.12, 95% CI: 3.12–5.71), illness duration ≤ 30 days prior to treatment initiation (AOR: 3.51, 95% CI: 2.17–5.13), supplementary food provision (AOR: 3.03, 95% CI: 1.90–4.83), meal frequency ≥ 3 times/day (AOR: 3.85, 95% CI: 2.41–6.14), good TB knowledge (AOR: 3.19, 95% CI: 2.19–4.05), and favorable attitude toward treatment (AOR: 2.41, 95% CI: 1.13–3.79).

**Conclusion:**

Approximately seven in ten adult TB patients achieved weight gain during the intensive treatment phase. Promoting drug adherence, early treatment initiation, nutritional supplementation, adequate meal frequency, and health education targeting TB knowledge and patient attitude are essential strategies for improving weight recovery and treatment outcomes.

## 1. Introduction

Tuberculosis (TB) is a preventable and curable infectious disease caused by *Mycobacterium tuberculosis*, the predominant species within the *Mycobacterium tuberculosis* complex (MTBC). Transmission occurs via airborne droplet nuclei of 1–5 μm in diameter, and susceptibility is determined by the host’s immune status, the degree of infectiousness, the degree of exposure, and surrounding environmental conditions [1–3]. TB affects individuals of all ages and both sexes; however, it is particularly concentrated among high‐risk groups, either due to a greater likelihood of progression upon infection or because they reside in environments where transmission risk is elevated [4, 5]. Globally, TB remains one of the leading causes of death from a single infectious agent, resulting in an estimated 1.25 million deaths in 2023, including 161,000 among people living with HIV [6]. In the same year, new TB cases per 100,000 population reached 134 per million, a 0.2% increase from 2022 with 87% of global cases concentrated in 30 high‐burden countries, with India, Indonesia, China, the Philippines, and Pakistan contributing 56%. Of those in 2023, 55% were men, 33% were women, and 12% were children and adolescents [3, 6]. Poor nutrition, low living standards, and financial hardship negatively affect treatment success and healthy weight gain [7]. Food insecurity contributes to weight loss during treatment and may result in catastrophic household costs exceeding 20% of annual income, far above the WHO End TB Strategy targets [1, 8]. Low socioeconomic status, limited healthcare access, and poor treatment adherence are associated with delayed recovery and inadequate weight gain [1, 3]. In sub‐Saharan Africa, including Ethiopia, constrained health services and suboptimal treatment quality often lead to insufficient weight gain during the first 2 months of therapy, which is a predictor of poor weight gain [2].

Undernutrition at diagnosis is strongly associated with poor weight gain, relapse, and increased mortality [3]. Inadequate weight gain during treatment should prompt evaluation for drug resistance, poor adherence, comorbidities, and unmet nutritional needs [9]. Early nutritional support, particularly within the first 2 months of treatment is critical, especially for patients nearing severe undernutrition [10]. Early changes in body mass index (BMI) are further influenced by sociodemographic factors including age, sex, income, education, living conditions, and dietary practices [11, 12].

TB is a significant global health issue, affecting millions of people each year [6]. Nutritional status, particularly weight gain during treatment, is critical for effective TB management [8]. Weight loss is a common symptom that leads to undernutrition and negatively affects recovery and treatment outcomes [5, 13].

During the intensive phase of TB treatment, weight loss can have serious consequences [14]. Patients who lose more than 5% of their body weight have a 2.5‐fold higher risk of death than those who maintain or gain weight [8]. Substantial weight loss is also strongly associated with treatment failure, with rates of up to 30% [10, 14].

Failure to gain weight in the first 2 months of treatment can delay treatment response, making patients 1.8 times more likely to remain infectious and contribute to TB transmission [15]. Nutritional counseling to increase protein, energy, and fat intake, along with micronutrient supplementation, has been shown to significantly improve weight gain during the intensive phase [10]. Severe weight loss further weakens the immune system and complicates recovery [16]. Weight gain is therefore a key indicator of successful TB treatment, though it varies according to socioeconomic status, treatment delays, nutritional support, and the quality of healthcare services [3]. According to WHO (2024), TB patients should aim to achieve a BMI of ≥ 18.5 kg/m^2^ within the first 2 months of treatment [15]. Adults typically gain approximately 2 kg during the intensive phase, reaching a BMI of around 19; however, patients with substantial pretreatment weight loss should also regain lean body mass to ensure full functional recovery [5, 17]. Evidence from India shows that 39% of TB patients gained more than 5% of their baseline weight and 50.2% gained over 10% [14]. A cross‐sectional study in Ghana’s Tema Metropolis found that 61% of TB patients were knowledgeable about nutritional‐related weight gain, and 65.8% had a favorable attitude toward weight gain [18]. In Ethiopia, Haramaya district, mean weight gain during the first 2 months of treatment was 1.79 kg, with significantly lower gains among people living with HIV compared to HIV‐negative patients [5, 8]. Delayed treatment initiation (> 30 days) and poor weight gain were associated with unsuccessful TB outcomes in studies from Horro Guduru Wollega Zone and Gibe District, Ethiopia, with a 14.5 poor outcome rate reported in the latter [9, 19]. Despite the growing evidence base, data on weight gain and its determinants in East Gojjam Zone, Northwest Ethiopia, remain scarce. This study, therefore, aimed to determine the magnitude of weight gain and its determinants among adult TB patients during the first two months of intensive phase follow‐up in public health institutions of East Gojjam Zone, Northwest Ethiopia, 2025.

## 2. Methods and Materials

### 2.1. Study Design, Setting and Period

Institution‐based cross‐sectional study design was conducted in public health institutions in the East Gojjam Zone, Amhara Region, Ethiopia. East Gojjam Zone has a projected population of 2,966,021 [20] and comprises 26 administrative areas, including 9 town administrations and 17 rural woredas. There are 11 hospitals, 105 health centers, and 435 health posts, all nonprofit public institutions. Debre Markos, the administrative town of East Gojjam, is located 265 km from Bahir Dar, the capital of the Amhara National Regional State, and 300 km from Addis Ababa, the capital city of Ethiopia. During the study period, a total of 116 public health institutions were providing TB DOTS services, comprising 105 health centers and 11 hospitals, which were collectively serving 1, 341 TB patients [20]. The study was conducted from June 4 to August 4, 2025.

### 2.2. Source and Study Populations

The source population comprised all TB patients aged ≥ 18 years receiving follow‐up during the intensive phase of treatment at public health institutions in the East Gojjam Zone, whereas study populations included all randomly selected TB patients aged ≥ 18 years attending TB clinics at selected public institutions during the study period. Patients who had completed their first 2‐month intensive phase follow‐up were included, whereas patients who were seriously ill at the time of data collection or who defaulted during the intensive phase follow‐up were excluded.

### 2.3. Sample Size and Sampling Procedure

The sample size was calculated using the single population proportion formula based on weight gain prevalence (*P*) of (57.8%) from a prior study conducted in Amhara region hospitals [21], with a 5% margin of error (*d*), a 95% confidence interval, a design effect of 1.5, and a 10% nonresponse rate. Thus, by considering all assumptions, the formula is as follows: *n* = (zα/2)^2^∗*p*(1−*p*)/*d* ^2^, yielding a final sample size of 619. The sample size was also calculated using the double population proportion formula, based on key exposure variables (latrine access, age, and family size) identified in a previous study from Haramaya District [21]. Therefore, using the EPI‐INFO 7 software and by considering the following assumptions, a 95% confidence interval, 80% power, an exposed‐to‐unexposed ratio, an adjusted odds ratio, and a nonresponse rate.

Finally, the largest sample size calculated was found to be 619 as compared to that in Table 1.

This study employed a multistage random sampling technique, requiring adjustments for a design effect. From the 116 total public health institutions, 31 were randomly selected comprising 7 hospitals and 24 health centers. The sample was allocated proportionally based on the projected number of TB patients for 2024–2025, and individual participants were selected using a lottery method from the TB registration logbooks.

### 2.4. Study Variables

**Dependent variable**: weight gain (Yes/No).

**Independent variables:** Socio‐economic and demographic factors **(**age, sex, religion, ethnicity, marital status, occupation, family income, educational status, and residence); treatment‐related factors (treatment category, drug adherence, and illness duration before treatment); dietary factor (meal frequency, supplementary feeding, dietary counselling, and dietary diversity score); comorbidities (HIV/AIDS, diarrhea, intestinal parasites, and asthma); support and supervision factors; and personal factors(TB knowledge and attitude).

### 2.5. Operational Definitions

#### 2.5.1. Weight Gain

Weight gain is an increase in patient body weight of ≥ 5% from baseline, measured at the end of the completed intensive phase of TB treatment [22–25]. Percentage of weight change was calculated as the difference in weights at the two measurements divided by the weight at the earlier time, multiplied by 100.

(1) Percentage  weight  change=weight at end−weight  at  startweight  at  start×100.

#### 2.5.2. Intensive Phase

Intensive phase is the first two months (56 days) of anti‐TB treatment [1].

#### 2.5.3. DOTS

DOTS is a directly observed short course treatment strategy and is a service of TB drug distribution to patients under the direct supervision of trained health professionals [1].

#### 2.5.4. Good Knowledge

It is measured using an eight‐item score, with ≥ 5 correct responses indicating good knowledge and those responses < 5 indicating poor knowledge [8, 10].

#### 2.5.5. Attitude Assessment Method

Respondents’ attitudes toward TB, its treatment, and associated weight gain were evaluated using Likert‐scale items of 11 questions. A score of ≥ 7 questions or higher out of 11 questions indicates a favorable attitude, while a score of < 7 questions suggests an unfavorable attitude. Participants’ responses were evaluated using a five‐point Likert scale (responses and scores: *strongly disagree* (Score 1), *disagree* (Score 2), *neutral* (Score 3), *agree* (Score 4), and *strongly agree* (Score 5) [26–28].

#### 2.5.6. Completed Drug Adherence

Completion of all prescribed doses during the 56‐day intensive phase without interruption [1].

#### 2.5.7. Adequate Dietary Diversity Score

Dietary diversity was assessed using 24‐h dietary recall across 12 food groups per FAO guidelines; scores above the mean were classified as adequate. The list of twelve food group items included the following: cereals, roots and tubers, vegetables, fruits, legumes. nuts and seeds, milk and milk products, eggs, meat and meat products, fish, sweets and sweet products, oils and fats, and spices and others [29].

#### 2.5.8. TB Category

Based on the revised definition given by WHO, patients were categorized as follows: classified as Category I (new cases) or Category II (previously treated) [1, 30].

### 2.6. Data Collection Methods and Quality Assurance

Structured questionnaires were adapted from existing literature, developed in English and translated into Amharic, and back‐translated into English to ensure consistency. The final Amharic version was used for data collection through TB clinic record reviews (extracting weight, BMI, HIV status, sputum type, TB category, and TB type) and interviewer‐administered questionnaires. Ten data collectors and four supervisors were assigned, with daily supervision to ensure data quality. One day of training was provided prior to data collection, and the questionnaire was pretested on 5% of the sample outside the study area.

### 2.7. Data Management and Statistical Analysis

Data were entered and cleaned using EpiData software Version 4.6 and exported to SPSS Version 26 for statistical analysis. Descriptive statistics, frequency tables, and graphs were used to summarize the data. Binary logistic regression was used to identify determinants of weight gain, with statistical significance set at *p* < 0.05. Variables with *p* < 0.25 in bivariable analysis were included in a multivariable logistic regression model using backward selection. Model fitness was assessed using the Hosmer–Lems how test (*p* > 0.05), and multicollinearity was checked using variance inflation factors (VIF < 10).

## 3. Results

### 3.1. Sociodemographic Characteristics of the Study Participants

A total of 619 adult TB patients participated in the study with a 100% response rate. The mean age was 37.94 (SD ± 15.1). Most participants were aged 25–34 years (27.6%) or > 45 years (29.9%), urban residents (58.5%), married (81.4%), Amhara (89.7%), Orthodox Christian (90.6%), and male (54.9%). The mean household size was 4.3 ± 1.7. Approximately 17.4% had college‐level education or above, 14.4% were government employees, and 68.3% received TB services at health centers (Table 1).

**TABLE 1:** Sample size determination for determinant factors using the double population proportion formula and the assumptions.

No	Variable	Proportion among exposed to unexposed group		Sample size	AOR (95% CI)	C.I at 95%	Power 80%	Design effect 1.5	Nonresp 10%	Total	Source
	Prevalence	57.8		**375**			**563**	**56**	**619**		
	Factors	P1 (%)	P2 (%)								
1	Latrine	38	56	262	2.14	(1.26, 3.65)	80	393	39	432	[8]
2	Family size	40	56	330	2.62	(1.43, 4.82)	80	495	50	545	[8]
3	Age	36	53	290	4.12	(1.36,12.51)	80	435	44	479	[8]

*Note:* P1 is the percentage of outcome in the exposed group. P2 is the percentage of outcome in the unexposed group. *R* is the ratio of nonexposed to exposed 1:1.

### 3.2. Clinical, Nutritional, and Comorbidity Factors

**FIGURE 1:**
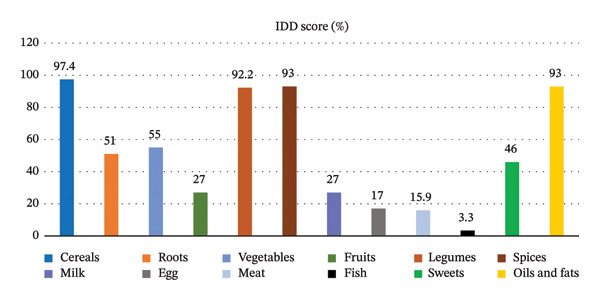
Magnitude of IDD score assessed over a 24‐h period based on FAO 2012, among adult TB patients during the first two months of the intensive phase follow‐up in East Gojjam Zone, Northwest Ethiopia, from June 4 to August 4, 2025. Bar chart titled “IDD Score (%)“ showing percentages for 12 food groups. Cereals (97.4%), legumes (92.2%), spices (93%), and oils and fats (93%) have the highest scores. Vegetables (55%), roots (51%), and sweets (46%) are moderate. Fruits (27%), milk (27%), egg (17%), meat (15.9%), and fish (3.3%) are the lowest. A data table alongside the chart lists the same values numerically.

Among clinical factors, 17.6% (*n* = 109) were smear‐positive pulmonary TB (PTB), 31.3% (*n* = 194) were smear‐negative PTB, and 51.1% (*n* = 316) were extrapulmonary PTB (EPTB). Most cases were new (79.2%, *n* = 490), with 16.6% (*n* = 103) relapses and 4.2% (*n* = 26) transfer‐ins. Of all participants, 61.7% (*n* = 382) initiated treatment within 1 month of illness onset, 69.1% (*n* = 428) achieved a BMI of ≥ 18.5 kg/m^2^, and 63.0% (*n* = 389) demonstrated completed drug adherence during the intensive phase. Regarding nutritional factors, daily meal frequency was once (8.4%), twice (26%), three times (6.3%), and four times (59.3%). Additionally, 61.8% received supplementary food, 71.2% received dietary counseling, and 38.4% experienced eating problems during treatment. The mean individual dietary diversity (IDD) score was 6.22 ± 1.50 (range, 1–12), assessed using a 24‐h dietary recall across 12 food groups among adult TB patients during the intensive phase. Using the mean as the cutoff point, 48.7% (302) had poor dietary diversity, while 51.2% (317) achieved good dietary diversity (Figure 1).

Among participants, 190 (30.6%) had chronic comorbidities: 97 (15.7%) were HIV‐positive, 52 (8.4%) had diarrhea, 27 (4.4%) had intestinal parasitic infections, 7 (1.1%) had asthma, and 7 (1.1%) had other conditions.

### 3.3. Supervision and Support‐Related Factors

Among participants, 66.3% (*n* = 411) received health education, and 54.9% (*n* = 334) received supportive supervision from health workers. Community support included material support 16.4% (*n* = 102), financial support 25.2% (*n* = 156), food support 65.6% *n* = 406), and regular psychological support (15.6%, *n* = 97).

### 3.4. Magnitude of Weight Gain

In this study, four hundred twenty‐eight (69.1%; 95% CI: 65.4, 72.7) respondents gained weight among adult TB patients during the intensive phase follow‐up (Table 2).

**TABLE 2:** Sociodemographic characteristics of study participants in East Gojjam Zone public health institutions, Northwest Ethiopia, 2025 (*n* = 619).

Variables	Category	Frequency	Percent
Age (years)	< 25	150	24.2
	25–34	171	27.6
	35–44	113	18.3
	≥ 45	185	29.9

Sex	Male	340	54.9
	Female	279	45.1

Marital status	Single	90	14.5
	Married	504	81.4
	Divorced	20	32.3
	Widowed	5	0.8

Ethnic groups	Amhara	555	89.7
	Oromo	40	6.5
	Tigre	12	1.9
	Others	12	1.9

Religion	Orthodox	561	90.6
	Muslim	31	5
	Protestant	15	2.4
	Catholic	7	1.1
	Others	5	0.8

Educational status	Not read and write	143	23.1
	Read and write	81	13.01
	Primary education	124	20
	Secondary education	163	21.3
	Graduated (college and above)	108	17.4

Occupation	Government employee	89	14.4
	Not government	530	85.6

Residence	Urban	362	58.5
	Rural	257	41.5

### 3.5. Factors Associated With Weight Gain

In the bivariable logistic regression analysis, nine variables with *p* values < 0.25 were selected for multivariable analysis: completed drug adherence, family size, educational status, illness of duration ≤ 4 weeks, supplementary feeding, meal frequency ≥ 3/day, dietary diversity score, favorable attitude, and good knowledge. Six variables remained independently associated with weight gain at *p* < 0.05 in the multivariable logistic regression model by using the backward elimination method (Table 2): Patients with completed drug adherence were 4.12 times more likely to gain weight than nonadherence patients (AOR: 4.12, 95% CI: 3.12–5.71); patients initiating treatment within 30 days of illness onset had 3.51 times higher odds of weight gain than those initiating later (AOR: 3.51, 95% CI: 2.17–5.13); patients receiving supplementary food had 3.03 times higher odds of weight gain (AOR:3.03, 95% CI: 1.90–4.83); patients consuming ≥ 3 meals per day had 3.85 times higher odds of weight gain (AOR: 3.85, 95% CI: 2.41–6.14); patients with good knowledge were 3.19 times more likely to gain weight than those with poor knowledge (AOR: 3.19, 95% CI: 2.19–4.05)); lastly, patients with a favorable attitude toward treatment 2.41 times higher odds of weight gain (AOR: 2.41, 95% CI: 1.51–3.85) (Table 3).

**TABLE 3:** Bivariate and multivariable logistic regression analysis of factors associated with weight gain among adult TB patients during the intensive phase, East Gojjam Zone, Northwest Ethiopia, from June 4 to August 4, 2025 (*n* = 619).

Variables	Weight gain (*n*)	Weight loss (*n*)	COR (95% CI)	AOR (95% CI)
Completed drug adherence	317	72	4.72 (3.13–6.44)^∗∗^	4.12 (3.12–0.5.71)^∗∗^
Interrupted drug taking (Ref)	111	119	1	1
Family size (≤ 4)	326	128	1.57 (1.17–2.46)^∗^	1.13 (0.77–1.87)
Family size (> 4) (Ref)	102	63	1	1
Educational level (literate)	341	135	1.62 (1.15–2.52)^∗^	1.3 (0.81–2.24)
Illiterate level (Ref)	87	56	1	1
Illness ≤ 4 weeks before Rx	309	73	4.19 (2.89–5.71)^∗∗^	3.51 (2.17–5.13)^∗∗^
Illness > 4 weeks [Ref]	119	118	1	1
Received supplementary feeding	297	86	2.76 (1.86–3.75)^∗∗^	3.03 (1.90–4.83)^∗∗^
Did not receive (Ref)	131	105	1	1
Meal frequency ≥ 3/day	328	78	4.75 (3.48–7.24)^∗∗^	3.85 (2.41–6.14)^∗∗^
Meal frequency < 3/day (Ref)	100	113	1	1
Good dietary diversity score	233	84	1.52 (1.12–2.23)^∗^	0.8 (0.54–1.37)
Poor dietary diversity (Ref)	195	107	1	1
Good TB knowledge	306	81	3.4 (2.35–4.78)^∗∗^	3.19 (2.19–4.05)^∗∗^
Poor Knowledge (Ref)	122	110	1	1
Favorable attitude of TB treatment	294	78	3.17 (2.13–4.30)^∗∗^	2.41 (1.51–3.85)^∗∗^
Unfavorable attitude (Ref)	134	113	1	1

*Note:* *P* value less than 0.01, Ref, reference category (1).

^∗^ *p* < 0.05.

^∗∗^ *p* < 0.01.

## 4. Discussion

The magnitude of weight gain among TB patients during the intensive phase in this study was 69.1% (95% CI: 65.4, 72.7). This finding is consistent with reports from similar low‐resource settings, including India [10] and Northwest Ethiopia [21, 31], suggesting that shared socioeconomic conditions and prevalent undernutrition influence patient outcomes. In contrast, the weight gain observed in this study was significantly higher than that reported in Amhara region, Northwest Ethiopia [31], which may reflect evolving access to health education, local initiatives, and temporal changes in care quality. Compared with the United States, where weight gain was reported in 31% of patients [32], demographic differences may be relevant; the mean age of participants in the US study was 38 years versus 37 years in ours, and younger patients may experience greater metabolic responsiveness to treatment [33]. Conversely, the weight gain in this study was lower than that reported in Malaysia (90%) [34] and Vietnam (96%) [35].

These higher rates likely reflect improved access to nutritional resources and more consistent intensive‐phase follow‐up care in those settings.

Treatment adherence was the strongest predictor of weight gain, with adherent patients being 4.12 times more likely to gain weight than nonadherent or interrupted patients. This finding aligns with findings from Wenago, Ethiopia [22, 36] and studies on TB adherence in Lesotho [37].

Early treatment initiation within 30 days of symptom onset had 3.51 times higher odds of weight gain compared to those who started treatment after 30 days (AOR: 3.51), consistent with evidence from Ethiopia [37, 38].), demonstrating that early treatment halts disease progression and accelerates immune and metabolic recovery [39].

Supplementary feeding was independently associated with weight gain; TB patients receiving nutritional supplementation were 3.03 times more likely to gain weight than those who did not (AOR: 3.03), consistent with evidence from Ethiopia and China [31, 39]. This reinforces the importance of integrating nutritional support into TB treatment programs. Meal frequency of ≥ 3 per day was also a significant predictor (AOR = 3.85; 95% CI: 2.41–6.14), consistent with research on meal frequency and nutritional status from China and multidisciplinary healthcare settings [40]. Patients with good TB knowledge were 3.19 times more likely to gain weight (AOR: 3.19), a finding consistent with studies from Ghana (62.6%) [18] and evidence on knowledge–behavior associations in TB [4, 11]. Finally, patients with a favorable attitude toward TB treatment and weight gain had 2.41 times higher odds of gaining weight, consistent with research from Ghana (65.8%) [18]. A favorable attitude promotes medication adherence and adequate nutritional intake and reduces bacterial load, thereby supporting weight recovery.

## 5. Conclusion

Approximately, seven in ten adult TB patients achieved weight gain during the intensive treatment phase, indicating a moderate level of nutritional recovery in the study setting. Six factors were independently and significantly associated with weight gain: completed drug adherence, early treatment initiation (≤ 30 days), supplementary nutritional support, adequate meal frequency (≥ 3/day), good TB knowledge, and favorable attitude toward treatment. Targeted public health interventions addressing these modifiable factors are essential to improve weight gain, treatment success, and overall patient outcomes in high‐burden TB settings.

## 6. Limitation of the Study

Since this study employed a cross‐sectional design, causal inference between drug use and weight gain cannot be established; furthermore, drug‐specific pharmacological correlations with weight gain could not be assessed, as all participants received the standardized first‐line HRZE regimen with limited pharmacological variability across the study population. Social desirability bias may have affected self‐reported adherence and dietary data.

## Author Contributions

Dires Delessa Alemu and Getnet Gedif conceptualized the study and developed the methodology. Software for data analysis was managed by Dires Delessa Alemu, Nakachew Mekonnen Alamirew, Habitamu Mekonen, and Yilkal Tafere. validation was conducted by Mequanente Dagnaw, Habitamu Mekonen, and Helen Asmamaw Asres. Formal data analysis was carried out by Dires Delessa Alemu and Getnet Gedif. The original draft of the manuscript was prepared by Dires Delessa Alemu and Nakachew Mekonnen Alamirew with critical review and editing with Yilkal Tafere, Habitamu Mekonen, Mequanente Dagnaw, and Helen Asmamaw Asres. All authors made significant contributions to this study. Getnet Gedif served as the corresponding author.

## Funding

The authors received no specific funding for this work.

## Disclosure

We confirm that this manuscript is an original work and has not been published previously, either in a whole or in part, in any journal or other publications. We also confirm that the manuscript is not currently under consideration for publication elsewhere and will not be submitted to another journal while under review by this journal.

## Ethics Statement

Ethical clearance was obtained from the research Ethical Review Committee at Debre Markos University’s College of Medicine and Health Sciences with the ref No. RCSTTD/437/01/117 dated March 03, 2025. A supporting letter was taken from the East Gojjam Zone health department. After explaining the study’s purpose, written verbal consent was obtained from all participants. Participation was voluntary, and confidentiality was maintained throughout the study. All the methods used during this study were conducted in accordance with the Declaration of Helsinki.

## Consent

The authors have nothing to report.

## Conflicts of Interest

The authors declare no conflicts of interest.

## Data Availability

All relevant data have been presented within the manuscript. The data set supporting the conclusions of this article is available from the corresponding author upon a reasonable request.
